# Supplementary figures and images for: Halophilic microbial community compositional shift after a rare rainfall in the Atacama Desert
Source: ISME J. 2019 Jul 4;13(11):2737–49. doi: 10.1038/s41396-019-0468-y (PMC6794293; doi:10.1038/s41396-019-0468-y)

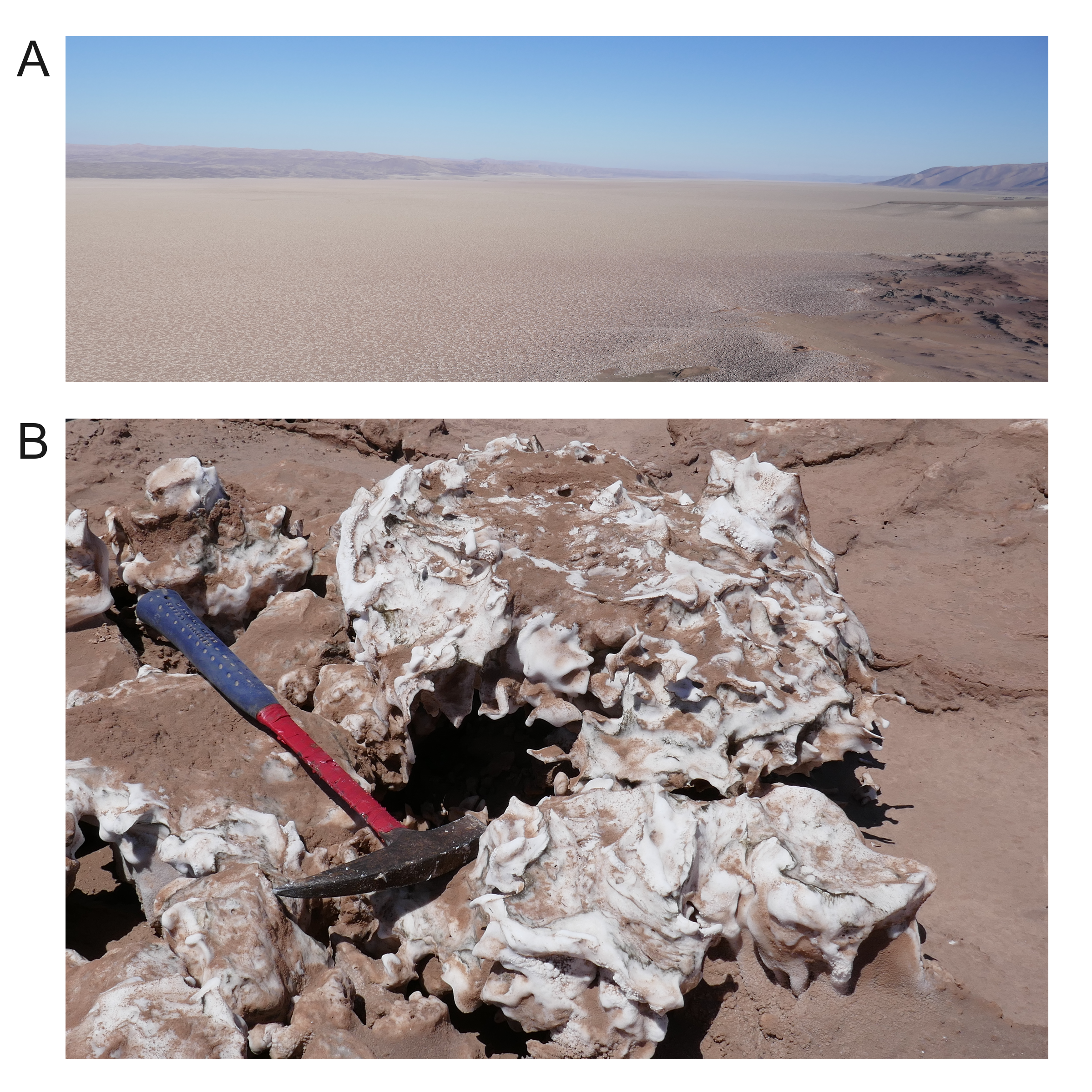

Supplement: Supplementary file 2 — Figure S1 [file 41396_2019_468_MOESM2_ESM.png]

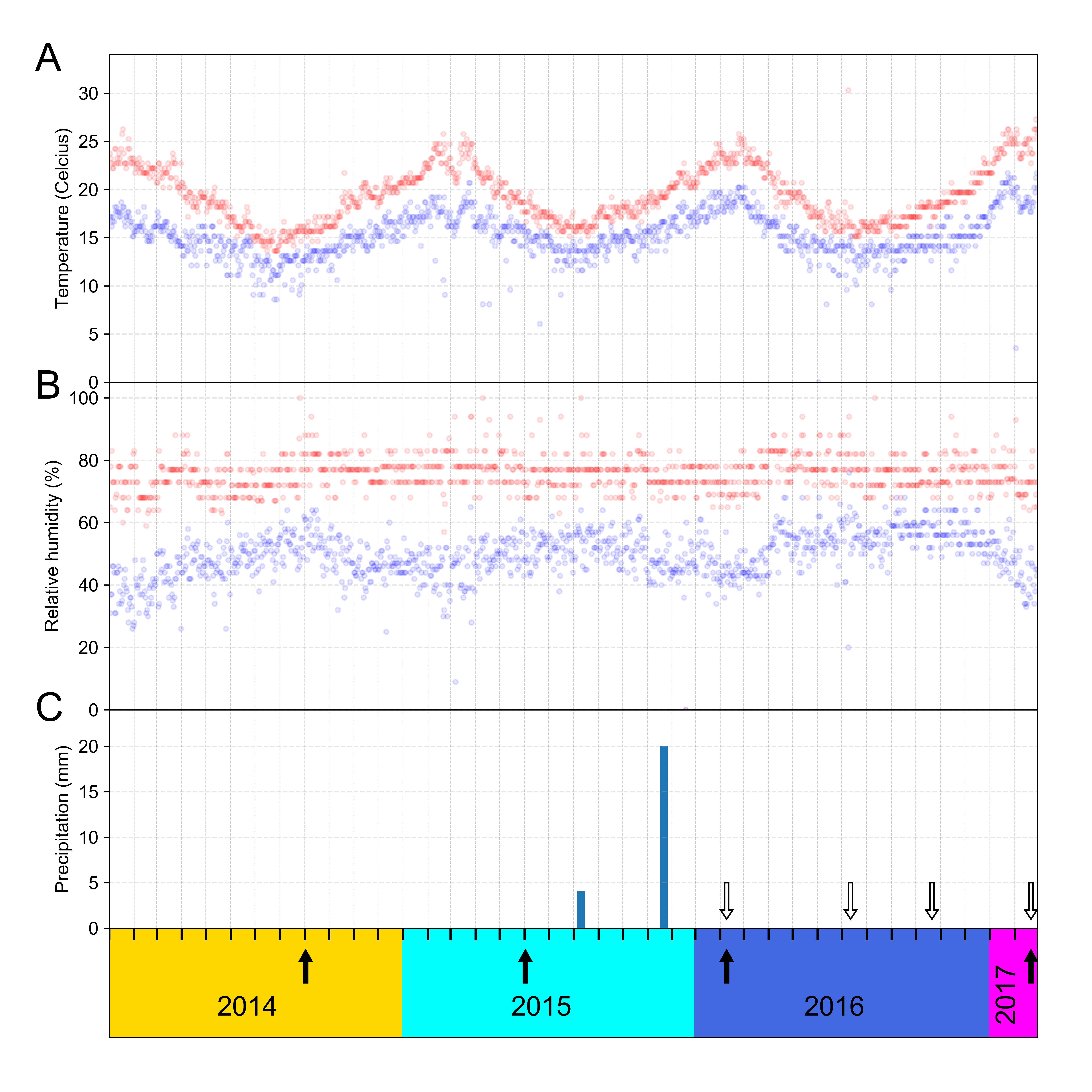

Supplement: Supplementary file 3 — Figure S2 [file 41396_2019_468_MOESM3_ESM.png]

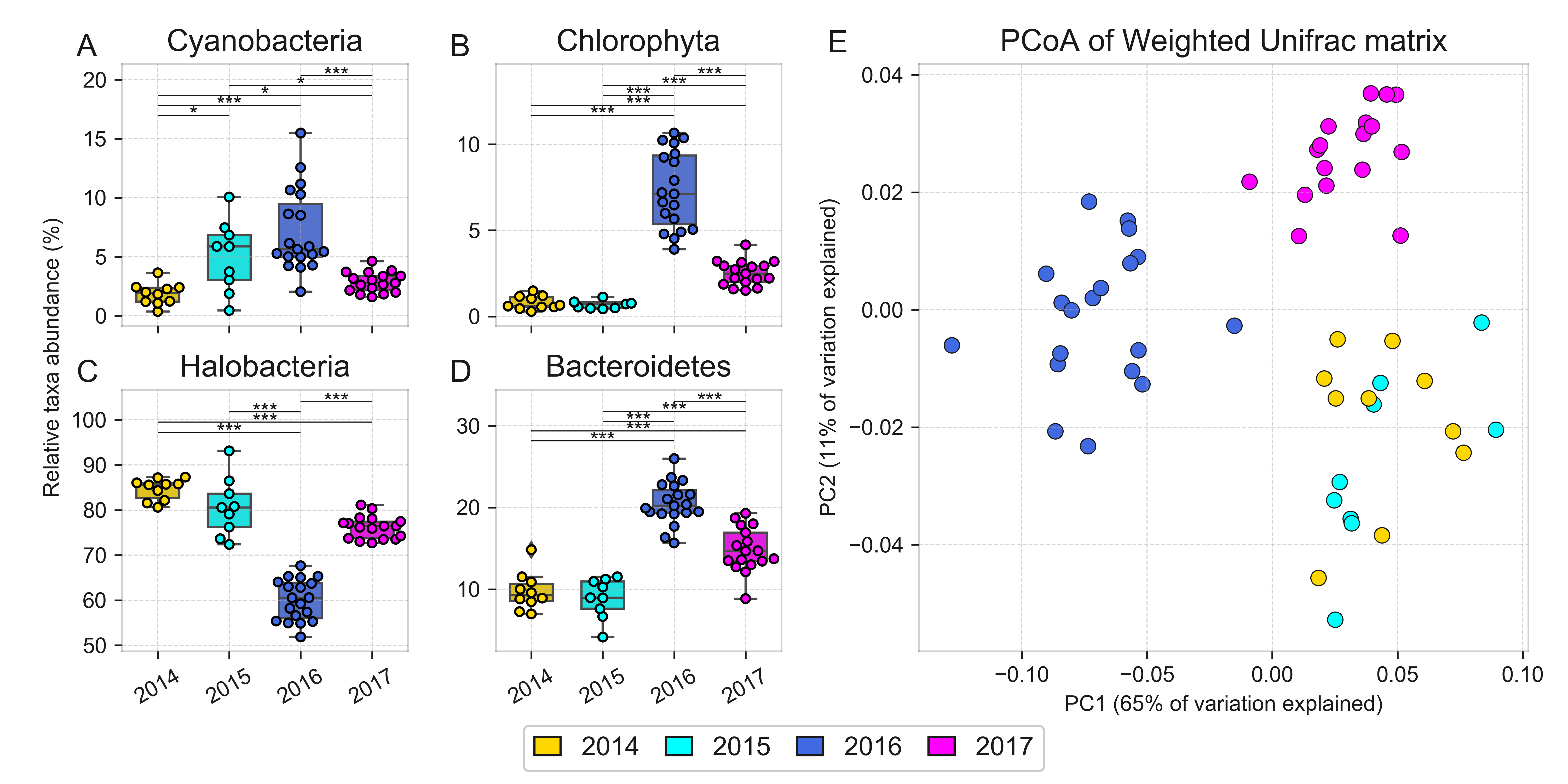

Supplement: Supplementary file 4 — Figure S3 [file 41396_2019_468_MOESM4_ESM.png]

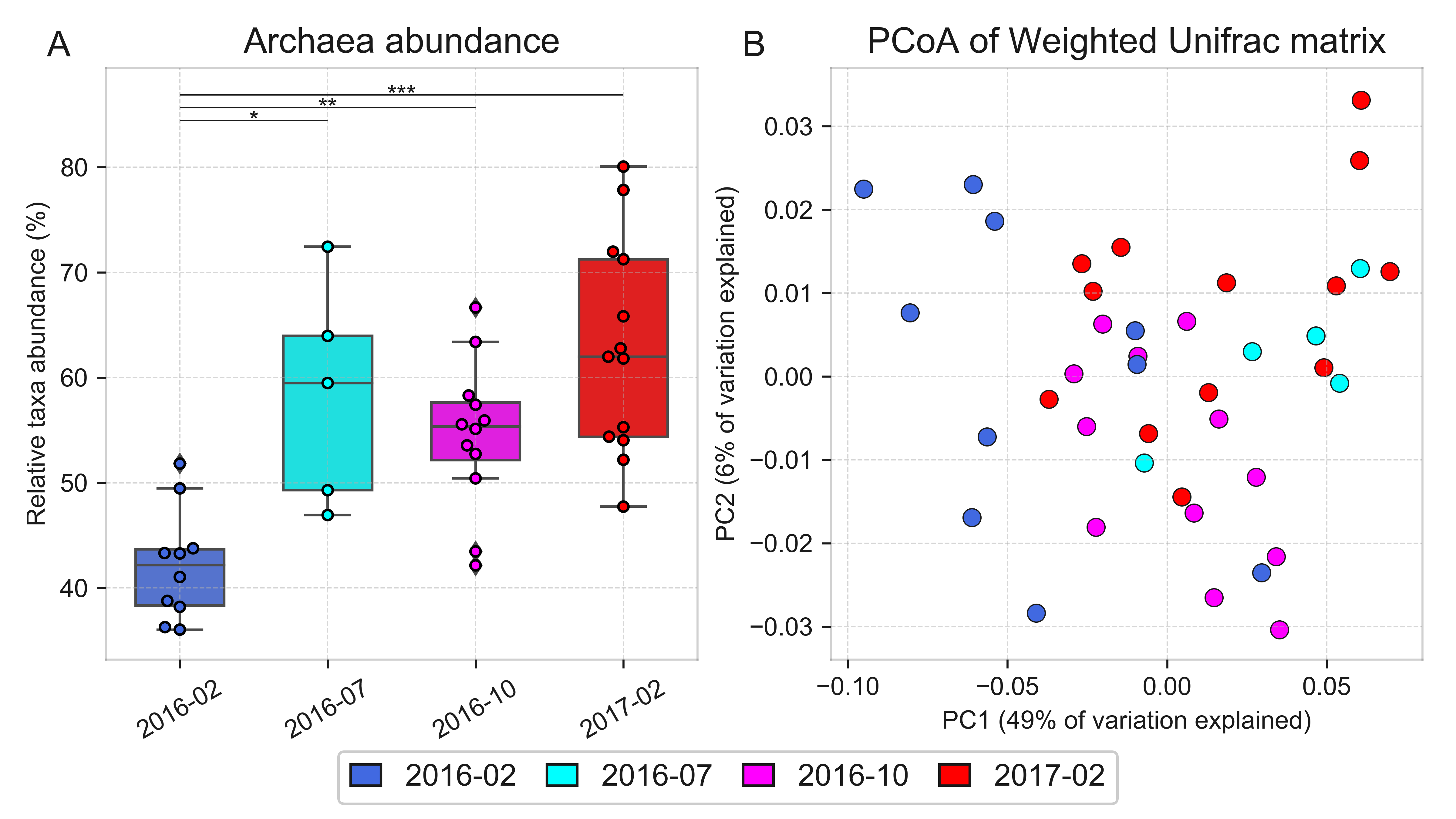

Supplement: Supplementary file 5 — Figure S4 [file 41396_2019_468_MOESM5_ESM.png]

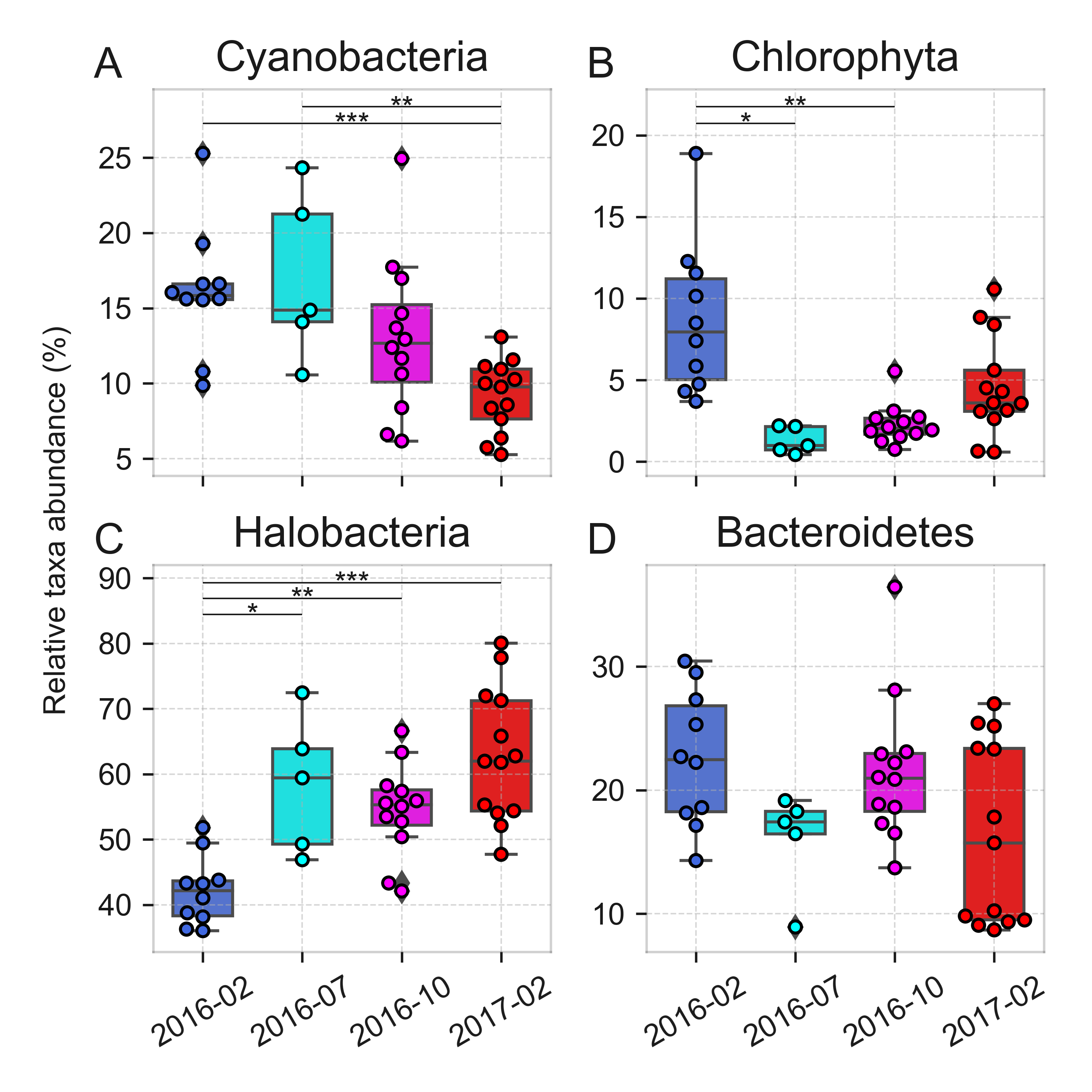

Supplement: Supplementary file 6 — Figure S5 [file 41396_2019_468_MOESM6_ESM.png]

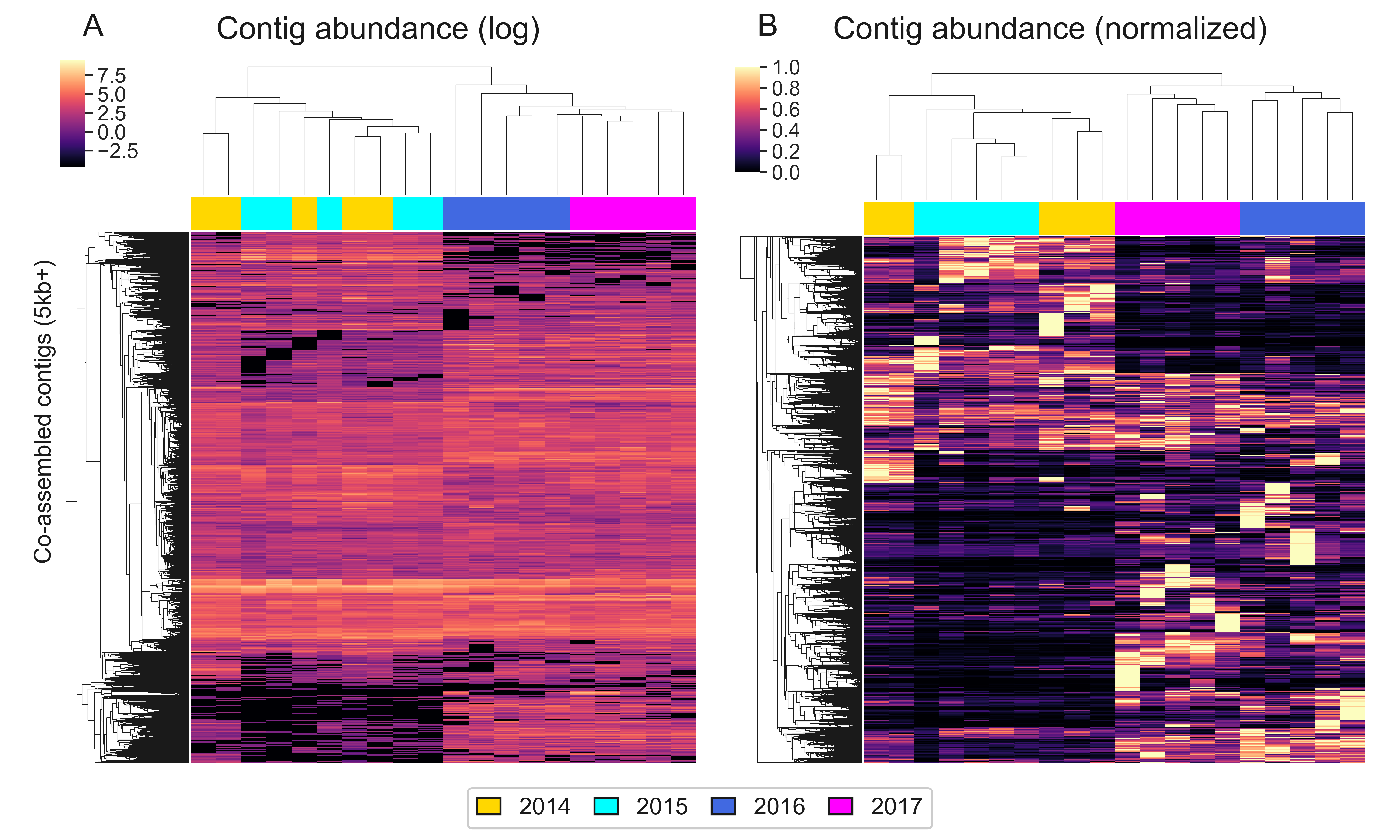

Supplement: Supplementary file 7 — Figure S6 [file 41396_2019_468_MOESM7_ESM.png]

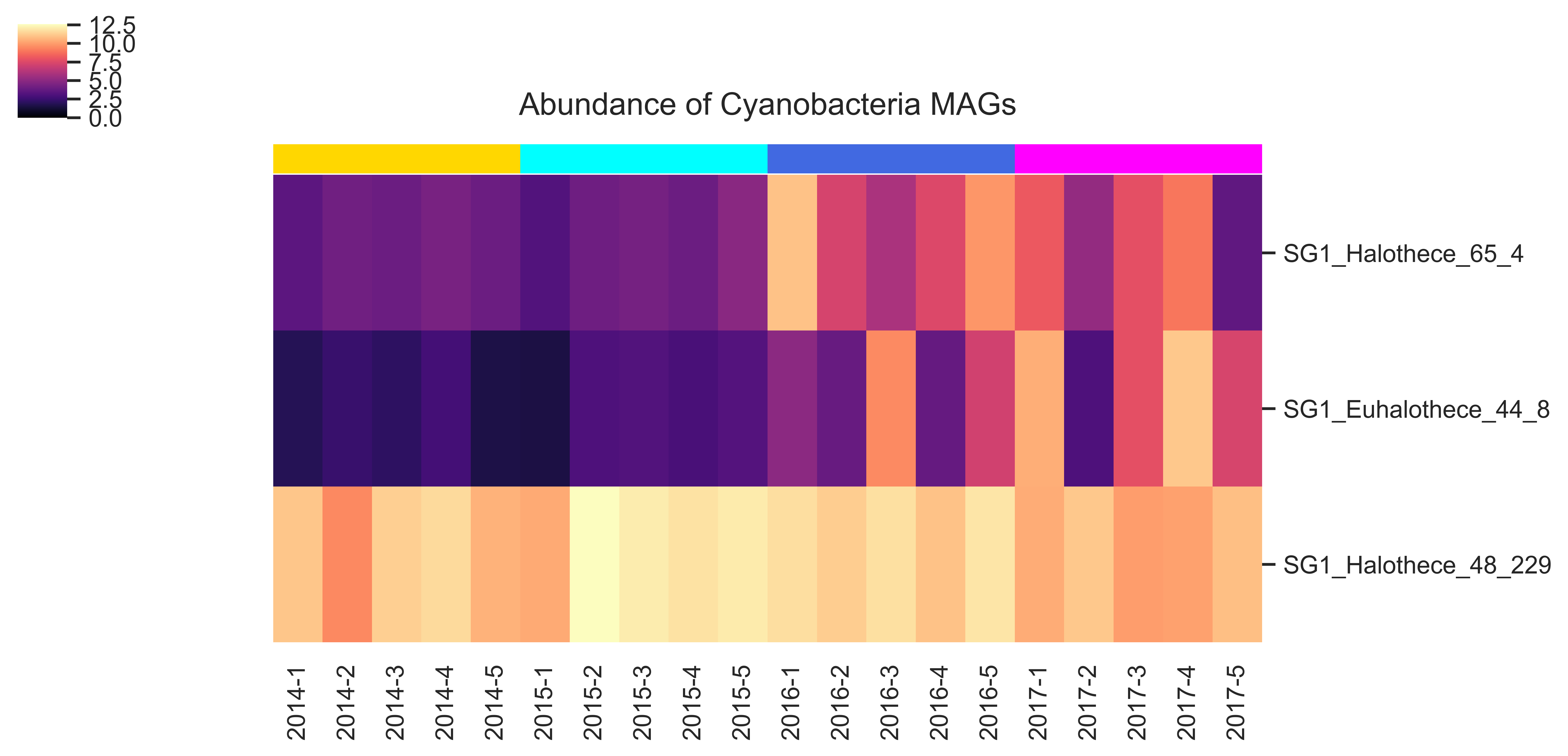

Supplement: Supplementary file 8 — Figure S7 [file 41396_2019_468_MOESM8_ESM.png]
